# P-439. Clinical And Therapeutic Characteristics Of Brucellosis Among Children

**DOI:** 10.1093/ofid/ofaf695.654

**Published:** 2026-01-11

**Authors:** Fatma Hammami, Khaoula Rekik, Amal Chakroun, Makram Koubaa, Fatma Smaoui, Chakib Marrakchi, Mounir Ben Jemaa

**Affiliations:** Infectious Diseases Department, Hedi Chaker University Hospital, University of Sfax, Tunisia, Sfax, Sfax, Tunisia; Infectious Diseases Department, Hedi Chaker University Hospital, University of Sfax, Tunisia, Sfax, Sfax, Tunisia; Infectious Diseases Department, Hedi Chaker University Hospital, University of Sfax, Tunisia, Sfax, Sfax, Tunisia; Infectious Diseases Department, Hedi Chaker University Hospital, University of Sfax, Tunisia, Sfax, Sfax, Tunisia; Infectious Diseases Department, Hedi Chaker University Hospital, University of Sfax, Tunisia, Sfax, Sfax, Tunisia; Infectious Diseases Department, Hedi Chaker University Hospital, University of Sfax, Tunisia, Sfax, Sfax, Tunisia; Infectious Diseases Department, Hedi Chaker University Hospital, University of Sfax, Tunisia, Sfax, Sfax, Tunisia

## Abstract

**Background:**

Brucellosis is a major zoonotic disease affecting individuals of all ages, with varied clinical presentations depending on the site of infection. Pediatric cases, though less frequently reported, require particular attention due to the diagnostic and therapeutic challenges. We aimed to study the clinical and therapeutic features of brucellosis among children.

**Methods:**

A retrospective study was conducted including patients aged ≤ 18 years hospitalized for brucellosis in the infectious diseases department between 2002 and 2024.

**Results:**

Thirty-one cases were identified, among whom 19 were males (61.3%). The mean age was 13 ± 4 years. Previous personal history of brucellosis and family history brucellosis were noted in 32.3% and 38.7% of cases, respectively. The main symptoms included fever (87.1%), night sweats (67.7%), and arthro-myalgia (61.3%). Splenomegaly and peripheral lymphadenopathy were each found in 9.7% of cases. Acute brucellosis accounted for 77.5% of cases. Complications included neurobrucellosis (3 cases), hip arthritis (2 cases), and sacroiliitis (2 cases). Wright’s serology was positive in all cases. Blood cultures were positive in 25.8% of the cases. Cerebrospinal or synovial fluid cultures were positive in 6.4% and 3.2%, respectively. Treatment included rifampicin (100%) combined with doxycycline (64.5%) or trimethoprim-sulfamethoxazole (35.5%). The disease evolution was favorable in all cases (100%).

**Conclusion:**

The diagnosis of brucellosis should be ruled out among children from endemic areas presenting with fever, night sweats and musculoskeletal symptoms. Prompt diagnosis and appropriate antibiotic therapy are key to ensuring favorable outcomes.

**Disclosures:**

All Authors: No reported disclosures

